# Advancements in the Heterologous Expression of Sucrose Phosphorylase and Its Molecular Modification for the Synthesis of Glycosylated Products

**DOI:** 10.3390/molecules29174086

**Published:** 2024-08-28

**Authors:** Hongyu Zhang, Leting Zhu, Zixuan Zhou, Danyun Wang, Jinshan Yang, Suying Wang, Tingting Lou

**Affiliations:** 1 Tianjin Key Laboratory of Food Biotechnology, College of Biotechnology and Food Science, Tianjin University of Commerce, Tianjin 300134, China; zhanghongyu@tjcu.edu.cn (H.Z.);; 2 Key Laboratory of Industrial Fermentation Microbiology, Ministry of Education, Tianjin Key Laboratory of Industrial Microbiology, College of Biotechnology, Tianjin University of Science & Technology, Tianjin 300457, China

**Keywords:** sucrose phosphorylase, transglycosylation, catalytic mechanism, heterologous expression, molecular modification

## Abstract

Sucrose phosphorylase (SPase), a member of the glycoside hydrolase GH13 family, possesses the ability to catalyze the hydrolysis of sucrose to generate α-glucose-1-phosphate and can also glycosylate diverse substrates, showcasing a wide substrate specificity. This enzyme has found extensive utility in the fields of food, medicine, and cosmetics, and has garnered significant attention as a focal point of research in transglycosylation enzymes. Nevertheless, SPase encounters numerous obstacles in industrial settings, including low enzyme yield, inadequate thermal stability, mixed regioselectivity, and limited transglycosylation activity. In-depth exploration of efficient expression strategies and molecular modifications based on the crystal structure and functional information of SPase is now a critical research priority. This paper systematically reviews the source microorganisms, crystal structure, and catalytic mechanism of SPase, summarizes diverse heterologous expression systems based on expression hosts and vectors, and examines the application and molecular modification progress of SPase in synthesizing typical glycosylated products. Additionally, it anticipates the broad application prospects of SPase in industrial production and related research fields, laying the groundwork for its engineering modification and industrial application.

## 1. Introduction

Glycosylation is a crucial biochemical process that produces compounds with diverse functions, enhancing the structural complexity, solubility, stability, and biological utilization of natural products [1]. This process is widely utilized in the food, cosmetics, and biomedicine industries. Current research focuses on synthesizing glycosylated compounds using low-cost, readily available raw materials. The main strategies for glycosylating organic small molecules include chemical and enzymatic methods. Chemical methods use toxic reagents, leading to environmental pollution, complex procedures, and many by-products [2]. Enzymatic methods are highly specific, do not require protection of non-target groups, operate under mild conditions, and are efficient and environmentally friendly [3]. Enzymatic catalysis demonstrates clear advantages in synthesizing glycosylated compounds. The carbohydrate-active enzymes (CAZy) database includes two main classes of glycosylation enzymes: glycosyltransferases (GTs) and glycoside hydrolases (GHs) [4,5]. GTs have broad substrate specificity and dominate most natural glycosylation reactions. However, the high cost of glycosyl donors limits large-scale applications [6]. GHs include glycosidases and transglycosidases, capable of hydrolyzing and synthesizing glycosidic bonds. They are widely distributed, utilize low-cost glycosyl donors, and have diverse applications [7].

SPase is a carbohydrate-active enzyme in the GH13 family of glycoside hydrolases, specifically the 18th subfamily, consisting of about 500 amino acids and a molecular weight of 50 to 60 kD [8]. It is mainly found in bacteria and other microorganisms, with small amounts in plant cells, functioning as monomers or homodimers [8]. SPase catalyzes the reversible conversion of sucrose and phosphate into α-D-glucose-1-phosphate and D-fructose through a covalent enzyme-sugar intermediate, the enzyme follows a double displacement mechanism [9]. SPase has wide substrate specificity, enabling phosphorylation, glycosylation, and hydrolysis with most sugar receptors [10], using monosaccharides or sugar alcohols as receptors, and catalyzing the production of oligosaccharides with an additional glucose unit, such as 2-*O*-α-d-glucopyranosyl-ɑ-d-glucose (kojibiose) [11] and 3-*O*-α-d-glucopyranosyl-d-glucose (nigerose) [12]. SPase acts on compounds with hydroxyl, carboxyl, and phenolic hydroxyl groups, synthesizing highly valuable products. For example, SPase can glycosylate and modify glycerol to synthesize 2-*O*-(α-d-glucopyranosyl)-sn-glycerol (aGG) [13,14] and modify phenolic substances like resveratrol [15], arbutin [16], phloretin [17], and catechin [18], enhancing the solubility, stability, and biological activity of polyphenolic compounds. Additionally, SPase can synthesize stable derivatives of l-ascorbic acids, such as 2-*O*-α-d-glucopyranosyl-l-ascorbic acid (AA-2G) [19,20]. Therefore, SPase has significant application potential in the food, pharmaceutical, and cosmetic industries.

Due to the complex metabolic regulation of wild-type strains, SPase production cannot meet industrial demands [21]. Hence, overexpressing SPase using genetic engineering techniques is essential for cost-effective industrial production. Additionally, SPase has a low affinity for certain receptor substrates (e.g., aromatic compounds), low catalytic efficiency, and requires enhanced thermal stability [22]. To improve the thermal stability of SPase, regional selectivity, and transglycosylation activity, protein engineering techniques such as directed evolution, semi-rational design, and rational design can be employed [23]. Directed evolution, also known as random mutation, mimics natural evolution through fragment recombination and random mutation [24]. In 1993, Frances Arnold [25] introduced the concept of directed evolution, incorporating recombinant fragments into target proteins via DNA recombination, error-prone PCR, and other technologies, leading to enzymes with enhanced specificity, activity, and tolerance, initiating a trend in non-rational protein design. Semi-rational design, based on the understanding of protein structure–function relationships, selects residues as target sites and saturates mutations at these sites to obtain improved variants [26]. Compared to directed evolution, semi-rational design provides faster modification and higher precision. Rational design, based on the structural and functional characteristics of the target protein, utilizes bioinformatics software for analysis followed by precise gene mutations and heterologous expression [27]. The advantages of rational design include fewer mutations, a higher likelihood of beneficial mutations, and a streamlined mutation library, significantly reducing the required workload and time [28,29]. It has been widely applied in designing various enzymes, antibodies, fluorescent proteins, protein vaccines, and more [30].

This paper initially outlines the primary source microorganisms of SPase, along with its three-dimensional structure and catalytic mechanism. It emphasizes the various heterologous expression systems of SPase, considering expression hosts and vectors. The discussion includes the purposes, strategies, and effects of molecular modification of SPase in the synthesis of various glycoside products. It also anticipates the extensive application prospects of SPase in industrial production and related research fields, aiming to provide references and a scientific basis for the basic research, applied research, and industrialization of SPase.

## 2. Source Microorganisms of SPase

In the 1940s, Kagan et al. [31] first identified SPase in *Leuconostoc mesenteroides*. Subsequently, researchers have discovered this enzyme in various other microorganisms, including *Clostridium pasteurianum, Pseudomonas saccharophila, Streptococcus mutans, Lactobacillus acidophilus, Bacillus megaterium, Bifidobacterium longum, Bifidobacterium adolescentis, Thermoanaerobacterium thermosaccharolyticum, Lactobacillus reuteri, Lactobacillus johnsonii, Bifidobacterium breve,* and *Bifidobacterium lactis* [8,32,33,34,35,36,37,38,39,40] (Table 1).

## 3. Structure and Catalytic Mechanism of SPase

### 3.1. Structure of SPase

**Figure 1:**
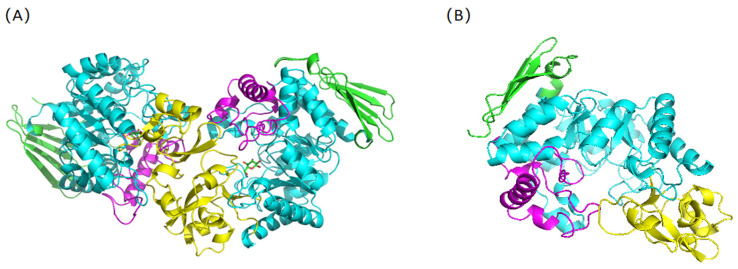
Crystal structure of *Ba*SP and *Lm*SP. (**A**) *Ba*SP(PDB:2GDV); (**B**) *Lm*SP (homology modeling based on 1R7A); Note: The image was generated using PyMOL (https://www.pymol.org/ accessed on 15 July 2024) where different colors represent distinct domains. Specifically, blue denotes Domain A, yellow signifies Domain B, purple represents Domain B’; and green indicates Domain C.

SPases from various sources exhibit distinct substrate specificities and protein structures. In 2004, Desiree Sprogoe et al. [53] elucidated the crystal structure of SPase (*Ba*SP) from *Bifidobacterium adolescentis*, revealing it as a homodimer, as depicted in Figure 1A. Conversely, SPase (*Lm*SP) from *Leuconostoc mesenteroides* is monomeric, as shown in Figure 1B [54]. The crystal structure of *Ba*SP comprises four domains: A (residues 1–85, 167–291, 356–435), B (residues 86–166), B’ (residues 292–355), and C [53]. The active sites, Asp192 (glucose anomeric carbon binding site) and Glu232 (glucosyl binding site) are situated at the C-terminal ends of β4 and β5 within domain A. Furthermore, domain A exhibits a (β/α)8 barrel structure, characteristic of the GH13 family [53]. The B domain consists of two short α-helices and two anti-parallel β-sheets, which mainly consist of a loop region (residues 130–140) and contribute to enzyme specificity. The B’ domain mainly consists of a loop region (residues 336–345) with one long and one short α-helix, which reduces the size of the substrate channel and hinders oligosaccharide binding. The C domain comprises five anti-parallel β-sheets with a 1,1,1,1 topology, typical of the glycoside hydrolase family [10].

### 3.2. Catalytic Mechanism of SPase

**Figure 2:**
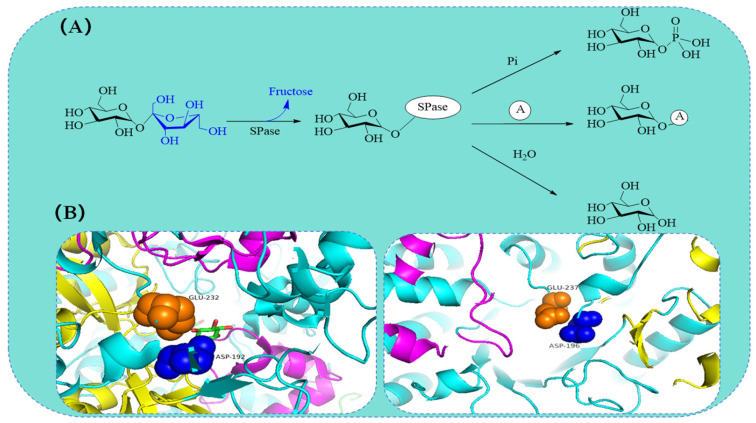
The catalytic mechanism of SPase. (**A**) Three catalytic reactions of SPase; (**B**) key amino acids of *Ba*SP and *Lm*SP. Note: Ⓐ can be glucose, vitamin C, glycerol, catechin, resveratrol, and other substrates.

The reactions catalyzed by SPase can be classified into three types: transfer between glucose units and phosphate (phosphorolysis and synthesis), hydrolysis, and transglycosylation [8]. During the reaction, SPase follows a double displacement mechanism, with specific carboxyl amino acid residues playing a crucial role in catalysis. For instance, Asp196 and Glu237 in *Lm*SP (right in Figure 2B), and Asp192 and Glu232 in *Ba*SP (left in Figure 2B) [55]. When sucrose enters the active site, the enzyme’s carbonyl residues attack the anomeric carbon of the glucose unit, forming a covalent β-glucosyl enzyme intermediate [56]. Apart from reacting with phosphate, glucosylated SPase can hydrolyze to produce α-D-glucose-1-phosphate or transfer the glucose unit to hydroxyl groups of receptor molecules, forming new α-D-glucoside products [8]. The glucose-enzyme intermediate may also be intercepted by water, resulting in irreversible hydrolysis of the donor substrate [8]. Under specific conditions, SPase can hydrolyze its donor substrate (sucrose or glucose-1-phosphate), and in transglycosylation reactions, recognize released glucose as the receptor substrate, forming glucosyl disaccharides, such as a mixture of kojibiose and nigerose [57], or a mixture of kojibiose and maltose [58]. Apart from glucose, other monosaccharides can also act as substrates for SPase. The mixed regioselectivity of SPase leads to the diversity of its products, so directing the synthesis of products with important application value through protein engineering is of great significance.

## 4. Heterologous Expression of SPase

With increasing demand for large-scale production, the low efficiency of wild-type strains in producing SPase poses challenges for industrial production. Genetic engineering to construct recombinant SPase-engineered bacteria is an effective method for achieving high enzyme expression levels. The *E. coli* expression system is favored for protein production due to its ease of cultivation, rapid growth, high expression levels, low production costs, stability, and wide applicability, significantly contributing to scientific research, and economic value. *E. coli* is employed as a host for the heterologous expression of SPase in most studies (Table 2).

In 1992, S. Kitao et al. [42] utilized a bacteriophage vector to express SPase from *Leuconostoc mesenteroides* in *E. coli*, achieving an enzyme activity of 55.7 U/mL, 80 times higher than that in *Leuconostoc mesenteroides*. Lee et al. [41] expressed SPase from *Leuconostoc mesenteroides* B-1149 in *E. coli* BL21(DE3), achieving a purified enzyme activity of 2.18 U/mg. Zhang et al. [50] expressed SPase genes from *Bifidobacterium longum* JCM1217 in *E. coli* BL21(DE3), achieving an activity of 122.1 U/mg after optimizing expression conditions, 1.85 times higher than before optimization, with a recovery rate of 86%. Yao et al. [21] utilized molecular chaperones pGro7 (GroES-GroEL), pG-KJE8 (DnaK-DnaJ-GrpE and GroES-GroEL), and pG-TF2 (GroES-GroEL-Tig) to promote soluble expression of SPase from *Thermoanaerobacterium thermosaccharolyticum* in *E. coli*.

However, the lack of post-translational modification capabilities in *E. coli* can lead to the inclusion of body formation, toxic effects on the host bacterium, and endotoxin production, requiring resolution in practical applications. In addition to *E. coli*, SPase is also expressed in *Bacillus subtilis*. Wang et al. expressed the SPase L341I_Q345S double mutant in *Bacillus subtilis* WS11, achieving significant extracellular enzyme expression with a crude enzyme activity of 5.3 U/mL [67]. *Bacillus subtilis* has many advantages as a host for heterologous protein expression: good safety, suitability for food and pharmaceutical industries, easy acquisition of exogenous DNA, genetic stability, a well-developed protein secretion system, and the ability to achieve high bacterial density in simple media, making it suitable for industrial production. However, this expression system also has disadvantages: at the end of the logarithmic phase of strain growth, secreted proteases may degrade the target protein, reducing protein yield and stability; plasmid stability is low and easily lost from *Bacillus subtilis* host cells; and the genetic manipulation system is not perfect.

The pET series vectors, developed by Studier et al. [68], are among the most widely used for SPase expression in *E. coli*. These vectors utilize the T7 RNA polymerase/promoter system with exogenous RNA polymerase. This vector contains the T7 promoter and T7 terminator, and when combined with the *E. coli* BL21 (DE3) strain, it can achieve efficient protein expression. Other SPase expression vectors from different sources also contain T7 promoters and systems similar to pET, except for the pQE30 vector, which has a T5 promoter (Table 2). When the exogenous RNA polymerase and promoter act together, they efficiently transcribe specific genes. The pET system can strictly control basal expression in the absence of inducers, effectively preventing the cloning of toxic genes. It has the lowest basal expression level. Promoter-encoded tags such as His-Tag provide affinity, facilitating subsequent purification. Numerous commercial vectors and host strains are available, simplifying selection. Specialized kits for directed cloning products make operations convenient and fast. Host strains are provided as competent cells, ready for immediate transformation.

## 5. Application and Molecular Modification of SPase

**Figure 3:**
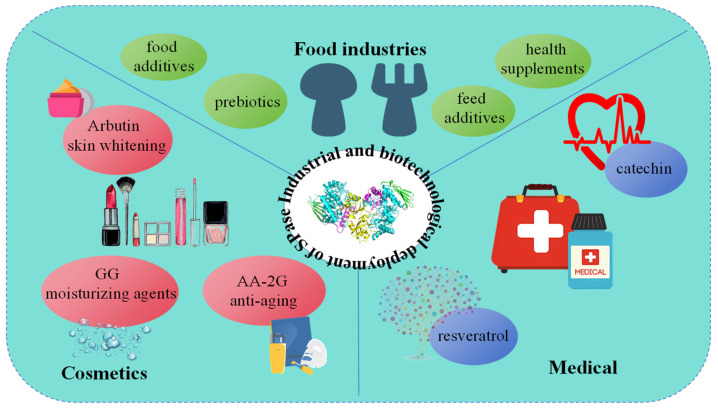
Application of products synthesized by SPase in the food, cosmetics, and the medical industry.

SPase is a glucosyltransferase capable of utilizing inexpensive donors and has broad substrate specificity for glycosyl receptors. The products synthesized via its transglycosylation have significant application potential in the food, cosmetics, and pharmaceutical industries (Figure 3).

However, natural SPase exhibits a low affinity for receptor substrates, leading to low product yields [22]. Additionally, the thermal stability of SPase requires improvement, and significant research efforts are focused on the molecular modification of SPase to enhance its application value. Protein engineering, using rational and semi-rational design strategies, has been widely applied to the molecular modification of SPase (Table 3).

### 5.1. Molecular Modification of SPase in the Synthesis of α-Arbutin

**Figure 4:**
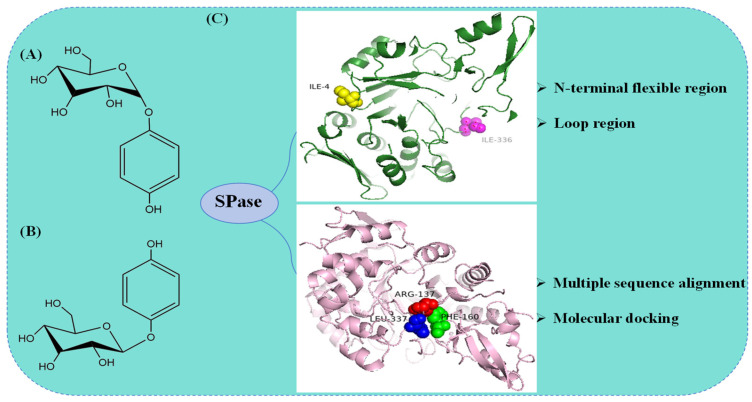
Structures of α-arbutin, β-arbutin, and molecular modification strategies of SPase in the synthesis of α-arbutin. (**A**) Structure of α-arbutin; (**B**) structure of β-arbutin; (**C**) semi-rational design strategy in the molecular modification of SPase for the synthesis of α-arbutin.

Arbutin, a natural glycoside of HQ [77], is primarily found in bearberry [78], cranberry [79], and pear plants [80]. Arbutin is an internationally recognized whitening and spot-removing agent that achieves its effect by significantly inhibiting tyrosinase. Tyrosinase is crucial in melanin synthesis; thus, arbutin effectively reduces melanin production by inhibiting tyrosinase activity. This property makes arbutin a vital raw material in the cosmetics industry [81]. α-Arbutin (Figure 4A) has been found to exhibit higher potency and better safety in inhibiting tyrosinase activity compared to β-arbutin (Figure 4B) [82]. These findings provide a scientific basis for innovating whitening products and offer crucial reference points for consumers in selecting these products.

The enzymatic synthesis mainly utilizes the transglycosylation activity of glycosyltransferases, resulting in a single, easy-to-extract, and environmentally friendly product, making it the primary method for producing α-arbutin. SPase demonstrates strong transglycosylation activity and broad acceptor specificity, using sucrose as an inexpensive and readily available substrate, thus showing significant potential and application value in the efficient production of α-arbutin.

In 1994, Kitao et al. [83] discovered that *Lm*SP could transfer the glucosyl group from sucrose to all 23 tested phenols and related compounds. It exhibited particularly high transglycosylation activity when HQ was employed as the substrate to produce α-arbutin, achieving a molar transfer rate of about 65% after optimizing the reaction conditions. Shen et al. [69] heterologously expressed SPase (*Sm*SP) from *Streptococcus mutans* UA159 in *Bacillus subtilis* WB600, utilizing recombinant *Bacillus subtilis* whole cells to catalyze α-arbutin synthesis. A semi-rational design strategy was employed to carry out site-saturation mutagenesis on the N-terminal flexible region and loop A region of *Sm*SP. By aligning the gene sequences of SPases from different sources, Pro2, Ile3, and Ile4 in the N-terminal region were chosen as mutation sites. Homology modeling revealed that the catalytic sites of *Sm*SP comprise Asp192, Glu232, Glu330, and Ile336. Glu330 and Ile336, which do not directly catalyze the substrate, were modified. Two positive mutants, *Sm*SPI4K and *Sm*SPI336L, were obtained, with α-arbutin yields of 61.1 g·L^−1^ and 71.7 g·L^−1^, respectively. By combining and catalytically validating these mutants, the single mutant *Sm*SPI336L was ultimately identified as the optimal mutant. Molecular docking between *Sm*SPI336L and HQ revealed that the formation of two new polar bonds upon binding increased the affinity of *Sm*SPI336L for HQ. After optimizing the reaction conditions, the yield of α-arbutin reached 110.3 g·L^−1^, with an HQ molar conversion rate of 88.7%, an increase of 2.74 times compared to the control (Figure 4C). Ao et al. [70] utilized *Lm*SP as a starting point, employing a semi-rational design approach based on multiple sequence alignment and molecular docking results to perform saturation mutagenesis on residues R137, F160, and L337 near the substrate binding site. Among them, the R137F mutant exhibited approximately 2.4 times lower affinity (K_m_) for the substrate HQ compared to the wild type, while the catalytic efficiency (k_cat_/K_m_) increased by 4.6 times. This is mainly due to the fact that the benzene ring of the HQ is partially accommodated by the hydrophobic pocket formed at the F137 site and the spatial site resistance was reduced. After optimizing the reaction conditions for the synthesis of α-arbutin, the final yield of α-arbutin reached 119.3 g·L^−1^ (Figure 4C). Su et al. [84] identified a novel SPase (PeSP) from *Paenibacillus elgii*, successfully characterized it, and applied it to α-arbutin biosynthesis. Optimizing PeSP reaction conditions resulted in an α-arbutin concentration of 52.60 g/L and an HQ conversion rate of 60.9%. Ao et al. [70] identified SPase mutants with high enzymatic activity established an anaerobic reaction system to prevent HQ oxidation, and enhanced cell robustness by knocking out the *lytC*, *sdpC*, and *skfA* genes in *Bacillus subtilis* to counteract the inhibitory effect of high HQ concentrations. After 24 h of fed-batch fermentation in a 5 L fermenter, the α-arbutin yield reached 129.6 g·L^−1^, laying the groundwork for large-scale industrial production.

### 5.2. Molecular Modification of SPase in the Synthesis of α-GG

**Figure 5:**
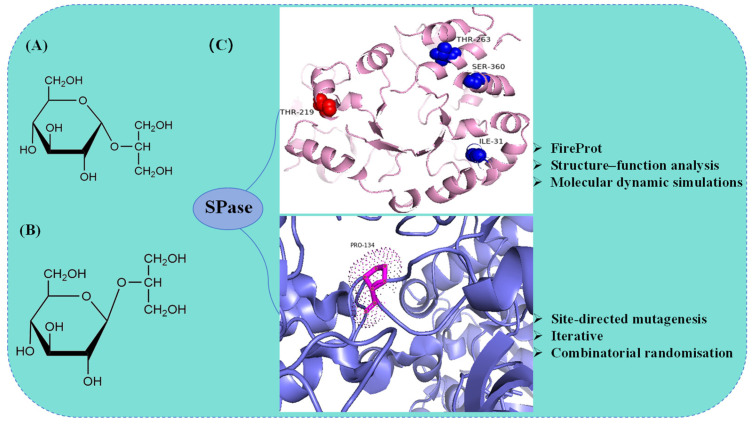
Structures of α-GG, β-GG, and semi-rational design strategies of SPase in the synthesis of α-GG. (**A**) Structure of α-GG; (**B**) structure of β-GG; (**C**) semi-rational design strategies of SPase for the synthesis of α-GG.

Glucosylglycerol (GG) exists in two forms: α-GG (Figure 5A) and β-GG (Figure 5B). GG was initially discovered in Japanese sake and koji-fermented foods [85]. It consists of one glucose molecule and one glycerol molecule. It has excellent osmotic regulation properties, protecting cells from high salt concentrations, extreme temperatures, and other environmental stresses [86]. Additionally, GG has potential prebiotic properties that promote probiotic growth, inhibit pathogenic bacteria, and enhance gastrointestinal resilience, indicating its potential as an effective functional food [87]. In the cosmetics industry, GG is valued for its outstanding moisturizing properties, with α-GG providing superior moisturizing effects compared to β-GG, making it an essential and effective ingredient in skincare products. GG also serves as a protein stabilizer, capable of maintaining protein structures [88]. Moreover, GG can act as a low-calorie sweetener and has the potential to prevent dental caries due to its non-cariogenic properties.

Current methods for preparing α-GG include chemical, microbial, and enzymatic synthesis [89,90]. Enzymatic synthesis of α-GG provides mild reaction conditions, high product purity, low-cost substrates, and high conversion rates. Consequently, enzymatic synthesis is more widely applied for α-GG production. Recently, molecular redesign through rational design has significantly increased α-GG yield, enhancing the application of enzymatic catalysis in large-scale α-GG production. Goedl et al. [91] cloned and expressed the SPase gene in *E. coli*, catalyzing α-GG synthesis using glycerol and sucrose with a 90% yield. Franceus et al. [71] used site-directed mutagenesis, iterative cycles of active site modification, and combinatorial screening to select the P134Q variant from 3900 variants of *Ba*SP, significantly enhancing its performance. Introducing a glutamine residue allowed the formation of hydrogen bonds with the hydroxyl group of glycerol and possibly with histidine at position 234. This variant exhibited a 21-fold increase in catalytic efficiency for glycerol and a 3-fold improvement in substrate regioselectivity (Figure 5C). Xia et al. [72] overexpressed *Lm*SP in *E. coli* and utilized the strategies of FireProt, structural-functional analysis, and molecular dynamics simulations to enhance its thermal stability. The single-point mutant T219L and the well-thermostable combinatorial mutant I31F/T219L/T263L/S360A (Mut4) were obtained. It is revealed that the transition of small polar amino acids to hydrophobic amino acids and the enhancement of amino acid hydrophobicity are key factors in improving protein stability. The crude enzyme solutions of mutants T219L and Mut4 at 40 U·mL^−1^ were incubated with sucrose and glycerol at 37 °C for 60 h, resulting in α-GG concentrations of 193.2 ± 12.9 g·L^−1^ and 195.8 ± 13.1 g·L^−1^, respectively, approximately 1.3 times higher than the wild type (Figure 5C). Kruschitz et al. [92] immobilized whole cells expressing the *Ba*SP mutant P134Q, achieving a maximum space-time yield of α-GG of 45 g·L^−1^·h^−1^ at a product concentration of 120 g·L^−1^. This demonstrated the feasibility of continuous production using a whole cell-derived solid SPase catalyst and provided strong technical support for the large-scale industrial production of α-GG.

### 5.3. Molecular Modification of SPase in the Synthesis of AA-2G

**Figure 6:**
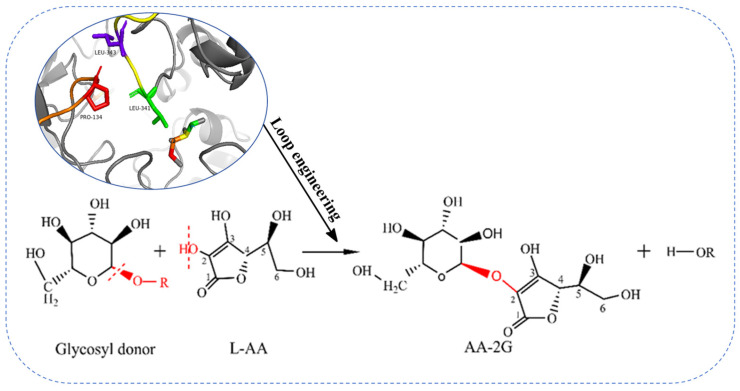
Loop engineering modification of *Ba*SP for the synthesis of AA-2G.

L-ascorbic acid (L-AA) is an essential micronutrient and co-factor with significant biochemical roles. However, L-AA is unstable under high temperatures and oxidative conditions, limiting its application [93]. AA-2G (Figure 6) is a highly stable form of L-AA, showing L-AA activity after hydrolysis by α-glucosidase in vivo, and is extremely stable ex vivo [94] AA-2G retains the biological activity of L-AA, enhances antibody production, promotes collagen synthesis in human dermal fibroblasts, and prevents acute skin inflammation caused by UV exposure [95,96]. Additionally, AA-2G is highly resistant to oxidative degradation caused by heat and ascorbate oxidase [97]. In the cosmetics industry, AA-2G is widely used in whitening products due to its potent melanin inhibition. Renowned cosmetic brands such as Shiseido, L’Oréal, and Lancôme incorporate AA-2G in their whitening product lines [98]. In addition to cosmetics, AA-2G, as an important supplement form of L-AA, is utilized as a food additive, showing promising applications in the food industry [99].

The chemical synthesis of AA-2G is challenging and is primarily achieved through enzymatic synthesis. Enzymes used in AA-2G synthesis include α-amylase [100], α-glucosidase [101], SPase [102], α-isomaltose glucosyltransferase [103], cyclodextrin glucosyltransferase (CGTase) [104], and glucan sucrose synthase [105]. In 2007, SPase from *Bifidobacterium longum* was first reported to convert L-AA and sucrose into AA-2G [93]. Compared to other enzymes, inexpensive sucrose can be used as a glycosyl donor and does not require glucoamylase treatment, SPase can offer significant advantages in AA-2G synthesis and great potential for application and development.

Gudiminchi et al. [102] heterologously expressed and purified SPase from *Bifidobacterium longum* for AA-2G synthesis and found that SPase glycosylation of L-AA showed significant pH dependence, with pH influencing regioselectivity. Under neutral conditions, a small amount of AA-6G is synthesized, but at pH 5.2, almost no AA-6G is produced. Therefore, during AA-2G synthesis, SPase produces mixed glycosylated products like AA-3G, AA-6G, and AA-2GG due to its low specificity and regioselectivity. Zhou et al. [39] modified two flexible loops (341LDLYQ345 and 133RPRP136) of SPase from *Bifidobacterium breve* (*Bbr*SPase), identifying P134, L341, and L343 as hotspots regulating loop flexibility. This led to the creation of the mutant L341V/L343F, which exhibited over 99% 2-OH selectivity for L-AA. Zhou et al. [106] also identified three impurities during AA-2G synthesis by *Bbr*SPase: AA-3G (impurity I), 2-*O*-α-d-glucopyranosyl-l-dehydroascorbic acid (impurity II), and 13-*O*-α-d-glucopyranosyl-2-*O*-α-d-glucopyranosyl-l-ascorbic acid (impurity III), proposing specific strategies to control each impurity. By fine-tuning the reaction conditions (pH = 5.5, T = 45 °C), the content of impurity I was reduced by 50%. During AA-2G purification, impurities were removed by applying low concentrations of alkali. Through semi-rational design and site-directed mutagenesis in the loop region, the mutant L343F was obtained, reducing impurities I and III by 63.9% and 100%, respectively, significantly lowering AA-2G production costs.

To address the poor thermal stability of SPase [107], Li et al. [40] heterologously expressed SPase from *Thermobacillus* sp. ZCTH02-B1 for AA-2G synthesis, achieving a yield of 39.94 ± 0.17 g·L^−1^, with the optimal reaction temperature being 65 °C and the optimal pH being 7.0, thus providing a new thermostable enzyme for AA-2G synthesis.

### 5.4. Molecular Modification of SPase in the Synthesis of Kojibiose

Kojibiose is a natural disaccharide consisting of two glucose molecules linked by an α-1,2-glycosidic bond. The unique α-1,2-glycosidic bond in kojibiose prevents its utilization by oral bacteria such as *Streptococcus mutans*, reducing acid production and potentially preventing dental cavities [108]. Kojibiose is also a low-calorie sweetener that enhances iron absorption [109]. Kojibiose is well-tolerated in the intestinal system and promotes the growth of Bifidobacteria, Lactobacilli, and Bacteroides, making it an excellent prebiotic component [110]. Additionally, kojibiose exhibits antitoxic activity by specifically inhibiting α-glucosidase I activity in various tissues and organs [111], facilitating the development of new drugs, particularly anti-HIV-I drugs [112].

**Figure 7:**
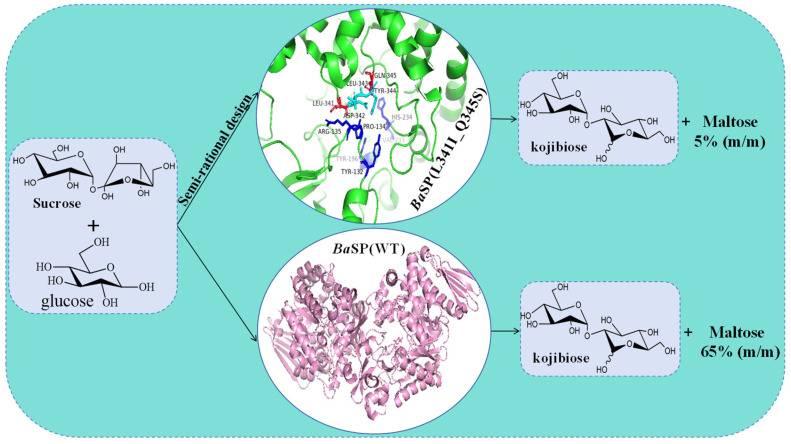
Semi-rational design of *Ba*SP for the synthesis of kojibiose.

Natural kojibiose is primarily found in molasses [113], beer [114], honey [115], and sake and starch hydrolysate [116]. However, the natural content of kojibiose is very low, making its separation and purification challenging. Currently, it is primarily prepared by chemical extraction and enzymatic methods. The chemical extraction method is cumbersome, producing numerous by-products and making separation and purification difficult, thus gradually being replaced by enzymatic methods. Enzymes used for synthesizing kojibiose include α-glucosidase [117], dextranase, β-galactosidase [118], and kojibiose phosphorylase [119]. Most of these enzymes have drawbacks such as expensive substrates, low yields, and numerous by-products. SPase can synthesize kojibiose using inexpensive sucrose as the donor and glucose as the acceptor through a transglycosylation reaction. It has been confirmed that SPase capable of synthesizing kojibiose is primarily derived from *Bifidobacterium adolescentis Ba*SP. In 2004, the elucidation of the *Ba*SP crystal structure further clarified the enzyme’s catalytic mechanism and center, and the discovery of the Loop region facilitated the semi-rational modification of SPase. During the synthesis of kojibiose by *Ba*SP, maltose is preferentially produced. To address the low regioselectivity of *Ba*SP, Verhaeghe et al. [119] employed a semi-rational design strategy to molecularly dock sucrose with the *Ba*SP structure, screening amino acid sites within 5 Å of the docking site. Using machine learning to explore the relationship between mutation sites and regioselectivity [120], they constructed a mutant library and obtained the double mutant *Ba*SP (L341I_Q345S) [119] with a kojibiose regioselectivity as high as 95% (Figure 7). Using this mutant, they established an efficient and scalable production process with a yield of 74%. Through simple yeast treatment and crystallization steps, high-purity kojibiose (purity > 99.5%) can be obtained. Beerens et al. [11] optimized kojibiose synthesis using various *Ba*SP variants and found that the L341I_Q345S double mutant remained active after one week at 55 °C, effectively synthesizing kojibiose, demonstrating good thermal stability. Process optimization increased kojibiose production to the kilogram scale. Using simple and efficient downstream techniques like yeast treatment and crystallization, high-purity kojibiose crystals (99.8%) were obtained. Wang et al. [67] successfully expressed *BaSP* in *Bacillus subtilis* and optimized the enzyme’s reaction conditions. Results indicated a significant improvement in recombinant enzyme expression, with a substrate conversion rate of 40.01%, kojibiose conversion rate of 104.45 g·L^−1^, and a selectivity of 97%. These findings laid the foundation for the industrial production of kojibiose using recombinant enzymes.

### 5.5. Molecular Modification of SPase in the Synthesis of Nigerose

**Figure 8:**
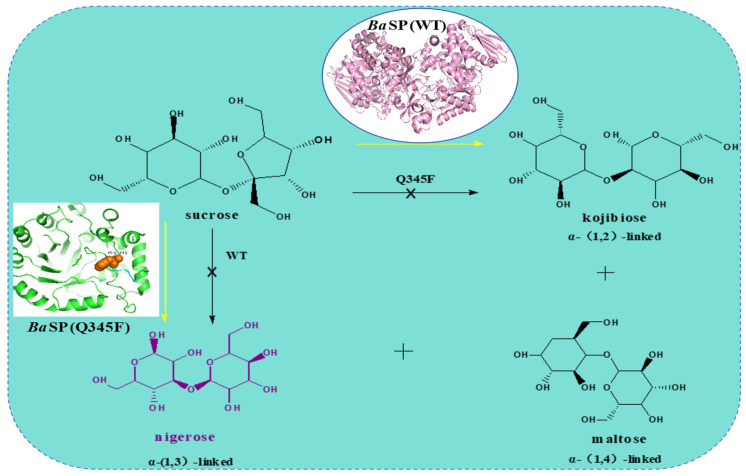
*Ba*SP (Q345F) for the selective synthesis of nigerose.

Nigerose is a rare disaccharide consisting of two glucose molecules linked by an α-1,3 glycosidic bond (Figure 8). Nigerose occurs naturally in the polysaccharides of *Aspergillus niger* [121], and has been found in Japanese rice wine and sake, previously known as sake sugar, enhancing the umami and aroma of sake and other fermented products. Its unique α-1,3 glycosidic bond prevents utilization by the oral microorganism *Streptococcus mutans* to produce acid, serving as a low-calorie sweetener to prevent dental caries [108]. Nigerose enhances immune activity in mice [122], serves as an additive in cryoprotective cell culture media [123], and acts as a proliferative factor for Bifidobacteria, Bacteroides, Eubacteria, and Lactobacilli, making it an excellent prebiotic component [108]. Additionally, nigerose is utilized in biocompatible nanocarriers for sustained drug delivery, showing potential for cancer treatment and antitumor activity [124]. Several methods for synthesizing nigerose have been reported, but obtaining high-purity nigerose remains challenging. Current methods generally require expensive raw materials or yield product mixtures that are difficult to separate. Nihira et al. [125] developed a method to synthesize nigerose from sucrose, but it requires a multi-enzyme system. In contrast, inexpensive sucrose can be utilized as a donor and glucose can be utilized as an acceptor by SPase to produce nigerose through a transglycosylation reaction [57].

In 1993, Kitao et al. [57] utilized sucrose and glucose as substrates to generate nigerose and kojibiose in a molar ratio of 1:2 via *Lm*SP. In 2016, Kraus et al. [12] reported that introducing the Q345F mutation altered the regioselectivity of *Ba*SP towards glucose. This variant no longer produces kojibiose but generates maltose and nigerose (Figure 8). Although the Q345F mutation enabled *Ba*SP to synthesize nigerose, its activity and regioselectivity were poor. This issue can be alleviated by adding dimethyl sulfoxide (DMSO) as a cosolvent, but DMSO’s toxicity limits the potential applications of nigerose in the food and pharmaceutical industries. In 2019, Jorick Franceus et al. [126] improved the activity of *Ba*SP for nigerose synthesis in an aqueous solution through a semi-rational design. The +1 subsite of *Ba*SP consists mainly of two highly dynamic loops that undergo key conformational changes during the catalytic cycle. One of these loops (341LDLYQ345) accommodates the Q345F mutation necessary for nigerose synthesis. Based on this, the remaining loop positions were individually targeted for NNK-based saturation mutagenesis. Among these, the double mutants D342G/Q345F and Y344Q/Q345F demonstrated moderate increases in activity and improved regioselectivity, raising the nigerose/maltose ratio by at least fourfold. Given the enhanced efficiency of the double mutants in nigerose synthesis, they were further combined to investigate if the resultant mutants could further enhance synthesis efficiency. The resulting triple mutant (D342G/Y344Q/Q345F) did not enhance regioselectivity further but exhibited a ninefold increase in transglycosylation activity for synthesizing nigerose compared to the Q345F mutant. Subsequently, the second flexible loop (132YRPR135) was targeted using the first-round triple mutant (D342G/Y344Q/Q345F) as a template. Two significantly beneficial mutations were identified at position 135, with the most effective being R135Y, which increased specific activity fourfold. The mutant libraries targeting other positions in the second flexible loop did not yield further improvements. Compared to the Q345F single mutant, the final mutant R135Y/D342G/Y344Q/Q345F enhanced the catalytic efficiency (k_cat_/K_m_) for nigerose synthesis by 68-fold.

### 5.6. Molecular Modification of SPase in the Synthesis of Polyphenol Glycosides

Polyphenolic compounds, including catechins, resveratrol, and flavonoids, exhibit significant antimicrobial and anticancer activities, indicating substantial potential in the medical field. Clinical studies have shown that resveratrol has promising potential in treating diabetes, cardiovascular diseases, and neurological disorders, although its low solubility limits its industrial applications. Glycosylation of resveratrol is a key research focus aimed at enhancing its solubility and medicinal properties. Dirks et al. demonstrated that the R134A mutant of SPase from *Thermoanaerobacterium thermosaccharolyticum* has a strong affinity for resveratrol, making it suitable for the quantitative production of resveratrol-3-*O*-α-d-glucoside in aqueous systems [76]. Kraus et al. [75] mutated the Q345 site of *Ba*SP, altering the enzyme’s spatial conformation through domain shift to accommodate a broader range of polyphenolic substrates. When resveratrol is used as the substrate, the yield of resveratrol-3-*O*-α-d-glucoside reaches 97% [12], significantly improving the solubility and bioavailability of resveratrol.

Studies have indicated that the glycosylation product (+)-catechin 3′-*O*-α-d-glucopyranoside (C-G) has superior water solubility and biological activity compared to catechin. Kitao et al. [127] successfully synthesized (+)-catechin 3′-*O*-α-d-glucopyranoside (C-G) by catalyzing catechin and sucrose with *Lm*SP, achieving a catechin conversion rate of 81%. Further studies confirmed that the *Ba*SP mutant Q345F can effectively glycosylate (+)-catechin, although it may produce region-specific isomeric mixtures, with C-G constituting only 51% [73]. Through semi-rational design, introducing Q345F/P134D, which has a strong tendency to form hydrogen bonds and low hydrophobic contact capacity, into the active site to replace aspartic acid, can effectively alter the orientation of flavonoids at the receptor catalytic site. This modification directs the glycosylation reaction towards generating C-G, achieving a C-G yield of 82% [58].

## 6. Conclusions

SPase catalyzes the reversible conversion of sucrose and phosphate into α-D-glucose 1-phosphate and D-fructose. Apart from reacting with phosphate, glucosylated SPase can hydrolyze to produce α-D-glucose or transfer glucosyl to hydroxyl groups in suitable receptor molecules to form new α-D-glucoside products. These unique catalytic properties endow SPase with extensive application potential in food, pharmaceuticals, and cosmetics, making it a focus of scientific research. However, SPase shows low affinity and product yield when catalyzing certain receptor substrates, and its thermal stability needs improvement. Consequently, numerous studies are focusing on rational, semi-rational, or irrational design strategies to modify SPase, aiming to enhance its substrate affinity, catalytic activity, and thermal stability, thereby increasing its industrial application potential.

This article systematically reviews the progress in the heterologous expression and molecular modification of SPase. Currently, the most reported hosts for SPase expression are *E. coli*, but *E. coli* tends to produce endotoxins, rendering it unsuitable for food industry applications. The molecular modification of SPase heavily relies on iterative or saturation mutagenesis and other semi-rational design methods. To enhance modification efficiency and reduce screening workload, more effective analytical and computational strategies should be applied to predict and construct virtual mutant libraries. The three-dimensional structure of SPase visually presents the enzyme’s active center and sites, which is crucial for guiding molecular modifications. However, to date, only the crystal structure of SPase from *Bifidobacterium adolescentis* DSM20083 has been elucidated.

Based on the discussion, we have summarized the following prospects and future trends. Firstly, *Bacillus subtilis* is recognized as a safe strain (GRAS, generally recognized as safe), with strong protein secretion capability and the advantage of not secreting endotoxins. It can be developed as an expression host suitable for industrial applications in the food industry. Secondly, research can focus on SPase substrate channels. Currently, research on the entry of substrate molecules into SPase’s active center and the exchange and release of products is limited. With the development of advanced molecular dynamics simulation technology, applying this technique to study SPase channel structures helps clarify substrate entry and product release processes, providing a theoretical basis for further SPase modification. Thirdly, the relationship between SPase’s hydrolytic and transglycosylation activities can be explored. SPase hydrolytic activity forms the basis of transglycosylation activity, the hydrolytic activity results in the formation of α-D-glucose-1-phosphate for the transglycosylation activity, but the exact relationship between hydrolytic and transglycosylation activities remains unclear. Fourthly, analyzing the structures of novel SPases from diverse sources provides resources for further application and molecular modification. In conclusion, this review provides valuable insights for researchers interested in further exploring the commercial applications of SPase.

## Figures and Tables

**Table 1:** Source microorganisms of SPase and synthesis of its products.

GenBank No.	Substrate	Product	Source Microorganisms	Ref.
AAO33821.1	Suc; Glu	kojibiose	*B. adolescentis* DSM20083	[34]
—	Suc	α-G-1-P	*P. saccharophila* ATCC9114	[38]
AY795566.1	Suc; Glu	kojibiose; nigerose	*L. mesenteroides* NRRLB-1149	[41]
BAA14344.1	Suc; Glu	kojibiose; nigerose	*L. mesenteroides* ATCC 12291	[42]
AAA26937.1	Suc	α-G-1-P	*Streptococcus mutans*	[37]
AGP78457.1	Suc	glucosylated(+)-catechin	*Alteromonas mediterranea*	[43]
AGK37834.1	Suc	levan	*Lactobacillus reuteri* LTH5448	[44]
—	Suc	α-G-1-P	*P. puterfaciens*	[45]
2207198A	Suc; Glu	kojibiose; nigerose	*L. mesenteroides* No. 165	[46]
AIE44773.1	Suc	α-G-1-P; α-glycosides	*T. thermosaccharolyticum*	[33]
CCA61958.1	Suc; phosphate	α-G-1-P; Fru	*Ruminococcus gnavus* E1	[47]
—	Suc	α-G-1-P	*Pseudobutyrivibrio ruminis*	[48]
—	Suc; phosphate	α-G-1-P	*L. mesenteroides* DSM 20193	[49]
AAO84039.1	Suc	Glu;Fru;by-products	*B. longum* SJ32	[35]
BAF62433.1	Suc	α-G-1-P	*B. longum* JCM1217	[50]
AAO84039.1	Suc; orthophosphate	Fru; α-G-1-P	*B. longum* SJ32	[51]
—	Suc	Glu; Fru	*B. longum* NCIMB 702259^T^	[52]

Note: —: No data reported. Fru is the abbreviation for D-Fructose; Glu is the abbreviation for Glucose; Suc is the abbreviation for Sucrose; α-G-1-P is the abbreviation for glucose-1-phosphate; *B.* is the abbreviation for *Bifidobacterium*; *P.* is the abbreviation for *Pseudomonas*; *L.* is the abbreviation for *Leuconostoc*; *T.* is the abbreviation for *Thermoanaerobacterium.*

**Table 2:** Recombinant expression of SPase in *E. coli* and *Bacillus.*

GenBank No.	Source Microorganism	Expression Host	Expression Vector	Ref.
2207198A	*L. mesenteroides* No. 165	*E. coli B*L21(DE3)	pET-28	[20]
A0A1Y3Q6Q6	*Thermobacillus* sp. ZCTH02-B1	*E. coli B*L21(DE3)	pRSFDute1; pET-5(+)	[40,59]
BAN03569.1	*Ilumatobacter coccineus* YM16-304	*E. coli* CGSC 8974	pET21a; pCXP34h	[33,59]
ADL69407.1	*T. thermosaccharolyticum* DSM 571	*E. coli* BL21(DE3)	pET21a	[60]
WP_094046414.1	*T. thermosaccharolyticum*	*E. coli* BL21(DE3)	pET20b+chaperone	[21]
BAF62433.1	*B. longum* JCM1217	*E. coli* BL21(DE3)	pET30	[50]
AAO84039.1	*B. longum* SJ32	*E. coli* JM109; E. coli BL21	p6×His119	[35,51,61]
BAA14344.1	*L. mesenteroides* ATCC 12291	*E. coli* BL21(DE3); *E. coli* M15	pET-28a; pQE30	[62,63]
AAN01605.1	*B. lactis*	*E. coli* JM109	pSuc	[64]
AAO33821.1	*B. adolescentis*	*E. coli* BW25113; *E. coli* BL21(DE3)	pYB1s; pCXP34h;	[65,66]
AAO33821.1	*B. adolescentis*	*Bacillus subtilis*	pBSMuL3	[67]
AGP78457.1	*Alteromonas mediterranea*	*E. coli* BL21(DE3)	pET28b	[43]
AGK37834.1	*Lactobacillus reuteri* LTH5448	*E. coli* JM109	pGEMTeasy	[44]
AY795566.1	*L. mesenteroides* NRRLB-1149	*E. coli* BL21(DE3)	pGEM-TEasy	[41]
2207198A	*L. mesenteroides* No. 165	*E. coli* JM109	—	[46]
AAA26937.1	*Streptococcus mutans*	E. coli RX4,	—	[37]
CCA61958.1	*Ruminococcus gnavus* E1	*E. coli* BL21(DE3)	pOPINE	[47]
—	*L. mesenteroides* DSM 20193	*E. coli* DH10B	pQE30	[49]
AAO33821.1	*B. adolescentis* DSM20083	*E. coli* XL1-Blue	—	[34]
BAF62433.1	*B. longum* JCM1217	*E. coli* BL21(DE3)	pET30	[50]
—	*B. longum* NCIMB 702259	*E. coli*	pSK	[52]

Notes: —: No data reported. *B.* is the abbreviation for *Bifidobacterium*; *P.* is the abbreviation for *Pseudomonas*; *L.* is the abbreviation for *Leuconostoc*; *T.* is the abbreviation for *Thermoanaerobacterium.*

**Table 3:** Molecular modification of SPase in the synthesis of glycosylated products.

GenBank No.	Source Microorganism	Mutant Site	Substrate	Products	Ref.
AAN58596.1	*Streptococcus mutans* UA159	I4; I336	HQ	α-arbutin	[69]
BAA14344.1	*L. mesenteroides* ATCC 12291	R137; F160; L337	HQ	α-arbutin	[70]
AAO33821.1	*B. adolescentis* DSM 20083	P134	Suc; glycerol	α-GG	[71]
BAA14344.1	*L. mesenteroides* ATCC 12291	T219; I31F/T219L/T263L/S360A	Suc; glycerol	α-GG	[72]
POO08785	*B. breve*	P134; L341; L343	Suc; l-ascorbic acid	AA-2G	[39]
AAO33821.1	*B. adolescentis* DSM 20083	Q345	Resveratrol; Suc	Resveratrol-3-*O*-α-d-glucoside	[73]
AAO33821.1	*B. adolescentis* DSM 20083	Q345	Suc	nigerose	[12]
AIE44773.1	*T. thermosaccharolyticum*	R134; H344	sucrose 6′-phosphate	α-G-1-P; Fructose 6-phosphate	[33]
AAO33821.1	*B. adolescentis* DSM 20083	Arg135; Leu343; Tyr344; Tyr132; Asp342; Pro134; Tyr196; His234; Gln345	Phosphate; Fru; D-Arabitol and pyridoxine	α-G-1-P; Fru; glycoconjugates	[66]
AAO33821.1	*B. adolescentis* DSM 20083	L341; D342; L343; Y344; Q345; Y132; P134; R135; Y196; V233; H234	Suc; Glu	kojibiose	[74]
AAO33821.1	*B. adolescentis* DSM 20083	Q345	Suc; polyphenols	resveratrol-3-α-d-glucoside and nigerose	[75]
ADL69407.1	*T. thermosaccharolyticum*	R134	Suc; polyphenol	Phloretin α-Glucosides and resveratrol-3-α-d-glucoside	[17,76]

Note: Fru is the abbreviation for D-Fructose; Glu is the abbreviation for glucose; Suc is the abbreviation for sucrose; α-G-1-P is the abbreviation for glucose-1-phosphate; B. is the abbreviation for *Bifidobacterium*; P. is the abbreviation for *Pseudomonas*; L. is the abbreviation for *Leuconostoc*, T. is the abbreviation for *Thermoanaerobacterium*, HQ is the abbreviation for hydroquinone.

## Data Availability

No new data were created or analyzed in this study.
